# A consensus statement on the renal monitoring of Australian patients receiving tenofovir based antiviral therapy for HIV/HBV infection

**DOI:** 10.1186/1742-6405-11-35

**Published:** 2014-11-10

**Authors:** Stephen G Holt, David M Gracey, Miriam T Levy, David W Mudge, Ashley B Irish, Rowan G Walker, Richard Baer, Jacob Sevastos, Riaz Abbas, Mark A Boyd

**Affiliations:** The Royal Melbourne Hospital and Faculty of Medicine University of Melbourne, 300 Grattan St, Parkville, Melbourne, VIC 3050 Australia; Royal Prince Alfred Hospital and Central Clinical School, Faculty of Medicine, University of Sydney, Missenden Rd, Camperdown, NSW 2050 Australia; Liverpool Hospital, Elizabeth St, Liverpool, NSW 2170 Australia; Princess Alexandra Hospital, 199 Ipswich Road, Woolloongabba, QLD 4102 Australia; Royal Perth Hospital, 197 Wellington St, Perth, WA 6000 Australia; Alfred Hospital, and Department of Medicine, Monash University, Melbourne, VIC 3004 Australia; Cairns Base Hospital, 165 The Esplanade, Cairns, QLD 4870 Australia; St. Vincent’s Hospital, 390 Victoria Street, Darlinghurst, NSW 2010 Australia; Medical Affairs, Gilead Sciences, Level 6, 417 St Kilda Road, Melbourne, VIC 3004 Australia; Kirby Institute, UNSW, Sydney, NSW 2052 Australia

**Keywords:** HIV, Hepatitis B, Tenofovir, Renal failure, Fanconi syndrome, Monitoring

## Abstract

A number of antiviral agents used against Human Immunodeficiency Virus (HIV) infection and hepatitis B virus (HBV) mono or co-infection have been associated with real nephrotoxicity (including tenofovir disoproxil fumarate (TDF), atazanavir, indinavir and lopinavir) or apparent changes in renal function (e.g. cobicistat, ritonavir, rilpivirine and dolutegravir). Patients with HIV are at higher risk of acute and chronic renal dysfunction, so baseline assessment and ongoing monitoring of renal function is an important part of routine management of patients with HIV.

Given the paucity of evidence in this area, we sought to establish a consensus view on how routine monitoring could be performed in Australian patients on ART regimens, especially those involving TDF. A group of nephrologists and prescribers (an HIV physician and a hepatologist) were assembled by Gilead to discuss practical and reasonable renal management strategies for patients particularly those on TDF-based combination regimens (in the case of those with HIV-infection) or on TDF-monotherapy (in the case of HBV-mono infection). The group considered which investigations should be performed as part of routine practice, their frequency, and when specialist renal referral is warranted. The algorithm presented suggests testing for serum creatinine along with plasma phosphate and an assessment of urinary protein (rather than albumin) and glucose.

Here we advocate baseline tests of renal function at initiation of therapy. If creatinine excretion inhibitors (e.g. cobicistat or rilpivirine) are used as part of the ART regimen, we suggest creatinine is rechecked at 4 weeks and this value used as the new baseline. Repeat testing is suggested at 3-monthly intervals for a year and then at least yearly thereafter if no abnormalities are detected. In patients with abnormal baseline results, renal function assessment should be performed at least 6 monthly. In HBV mono-infected patients advocate that a similar testing protocol may be logical.

## Review

### Introduction

Human immunodeficiency virus (HIV) infection has now become a chronic disease for most patients treated with antiretroviral therapy (ART) [1]. The improvement in prognosis has however unmasked a vulnerability to lifestyle and metabolic diseases like diabetes and hypertension, and has highlighted the potential for rare side effects of ART, including the nephrotoxic potential of some drugs [2]. HIV-infected individuals have higher risks of both acute and chronic kidney disease (CKD) [3–6] than the general population. Thus the monitoring of renal function and identification and treatment of risk factors for CKD are important elements of HIV management. For most patient groups, this can be done in line with existing guidelines, such as the Kidney Health Check [7]. However in the monitoring of patients on some antiretroviral agents there are key differences to this routine review. A recent Australian study found that there is room to improve the management of renal disease in HIV-infected patients [8].

Since its introduction in Australia in 2002, tenofovir disoproxil fumarate (TDF) has become the most widely used antiretroviral agent in HIV-infected patients and is listed as a preferred ART regimen in major guidelines [9–11]. Although preclinical studies and early clinical data did not identify any renal safety issues for TDF, a series of case reports and large population studies have suggested it has nephrotoxic potential in some HIV-infected patients, albeit with a low frequency [12–15]. Monitoring patients in order to pick up nephrotoxicity and proactive dose adjustment form part of routine HIV care. However, national and international guidelines lack detailed guidance for clinicians in the timing and nature of such monitoring (European AIDS Clinical Society [16] (EACS), British HIV Association [17] (BHIVA), US DHHS United States department of Health and Human Services [18]).

An expert panel convened by Gilead in Australia was set up to advise on the practical aspects of monitoring ART renal function in the absence of hard evidence. This took the form of a number of meetings where the evidence for nephrotoxity was sought and presented by various members of the panel (all of whom appear as authors of this paper) and based upon this a consensus monitoring algorithm was synthesised.

### Tenofovir

The prodrug tenofovir disproxil fumarate (TDF) is given because tenofovir (TFV) has poor oral bioavailability, but once absorbed TDF is rapidly hydrolysed to TFV. TFV is a potent nucleotide analogue reverse transcriptase inhibitor with activity against HIV and hepatitis B virus (HBV). Circulating TFV is taken up by endocytosis in most cells (except in the renal tubule where specific transporters are present) and rapidly phosphorylated further forming a nucleoside analogue that causes chain termination in HIV reverse transcriptase or HBV DNA polymerase [19]. The volume of distribution is within total body water, and TFV is mainly renally excreted with little hepatic metabolism. Of note TFV excretion exceeds glomerular filtration rate (GFR), so tubular secretion is thought to contribute 20–30% to its elimination.

A new formulation of TFV, tenofovir alafenamide (TAF, formerly GS-7340), is currently being evaluated in Phase III clinical trials. TAF is more stable in plasma and is predominantly hydrolysed to TFV intracellularly by cathepsin A in lymphocytes and macrophages. This results in high intracellular levels of the active phosphorylated tenofovir but reduced plasma levels of TFV. A recent phase II study showed significantly smaller changes in estimated creatinine clearance, renal tubular proteinuria than TDF [20].

### Evidence for renal effects

In the early randomised controlled trials of TDF vs. thymidine analogues in HIV [21–23] and subsequent follow-up studies there were small but statistically insignificant differences in renal function but no signals suggesting nephrotoxicity [12, 24–26]. However these studies involved relative healthy subjects and relied on measured changes in the serum creatinine (SCr). A series of case reports, predominantly of Fanconi syndrome started appearing in 2002 [27] and, propelled a re-examination of the renal safety data. The Viread Expanded Access Program [12] involving more than 10,000 people reported serious adverse renal events in 1.5 individuals per 1000 patient-years. Various cohort studies have subsequently reported associations between TDF exposure and small but statistically significant declines in estimated glomerular filtration rate (eGFR) over time. A recently published large cohort study of 22,603 HIV-infected persons [28] with a median follow up of 4.5 years (after 1January 2004) found that cumulative TDF, ritonavir-boosted atazanavir and ritonavir-boosted lopinavir use were all independent predictors of chronic renal impairment (confirmed eGFR of ≤70 mL/min) however, cumulative TDF use was not a significant predictor of CKD (eGFR of ≤60 mL/min). In a follow up publication expanded to over 35,000 patients with 200,119 person years of follow up 0.4% developed advanced renal failure (eGFR < 30) and 0.06% developed end-stage renal failure [29]. Further, the data showed that whilst TDF was discontinued as GFR fell, current or previous drug use did not predict these outcomes and only traditional risk factors and CD4 count were associated with increased risk. In a meta-analysis of 11 studies [13] there was an estimated mean reduction in eGFR of 3.92 mL/min among the TDF recipients compared with control subjects. Although this reduction was statistically significant, the magnitude was modest in clinical terms. This analysis also found no evidence that TDF use led to increased risk of CKD, severe proteinuria, hypophosphataemia or fractures. However, a large retrospective US cohort of 10,841 HIV-infected subjects [14] reported a small but significant increase in the relative risk of CKD (HR 1.44, 95% CI 1.3-1.6) and proteinuria (HR 1.30, 1.22-1.37) with TDF exposure, that was greatest in those with more than 3 years of exposure suggesting that monitoring of renal function should continue long-term.

Labarga *et al.* [30] reported that over time, subclinical proximal tubular dysfunction can develop, involving relatively low level proteinuria and phosphaturia, but in the absence of an impaired GFR. This occurred in up to 22% of closely monitored HIV patients treated with TDF. It was interesting to note that tubular dysfunction was also seen in 12% of ART-naïve patients. Although this dysfunction did not appear to have clinical sequelae it is nevertheless of concern not only for renal function but also potentially for future bone health.

TDF is associated with Fanconi syndrome (proteinuria, hypokalaemia, hypophosphataemia, phosphaturia, aminoaciduria and glycosuria) or acute kidney injury (AKI). Based on the available data, the frequency of Fanconi syndrome/AKI associated with TDF in HIV-treat patients is < 1% [12, 13]. In the expanded access program, a serious adverse renal event of any type was observed in 0.5% of patients, with failure (acute and chronic) reported in 0.3% and Fanconi syndrome in <0.1% [12]. In the meta-analysis the estimated increased risk of AKI was 0.7% [13].

Importantly, in many cases the renal dysfunction is fully reversible, and if detected early more likely to do so whilst if ignored could lead to dialysis requirements. Jose *et al.* reports that if detected TDF toxicity appears reversible in around half of cases within a year but recovery can be prolonged and may continue for over 5 years [31]. In this study between ~7-28% of patients did not fully recover, depending on the definition of full recovery. This study also showed that those with the largest change in GFR at discontinuation had a higher baseline GFR, and the duration of TDF treatment were the best determinants of incomplete recovery, making it important that initial and subsequent GFRs are recorded and compared [31]. No studies have fully addressed the concept of a TDF dose reduction in order to improve renal function and we do not endorse this strategy at present. Patients who have evidence of progressive nephrotoxicity, which only partially reverses with cessation of TDF, probably should not go back on this drug.

A South-African study has reported the association between nephrotoxicity (any decline in renal function from baseline) or death and baseline renal function [32]. They reported TDF nephrotoxicity in 2.4%, and those at highest risk of death were those with pre-existing renal dysfunction. Those who were switched from other regimens onto a TDF-containing regimen were also at highest risk of nephrotoxicity.

### Pathophysiology of renal injury

In the proximal tubules, high intracellular concentrations of TFV are thought to disrupt mitochondrial function affecting their number, size, shape and internal morphology [33]. TFV has a low inhibitory effect on mitochondrial DNA (mtDNA) polymerase-γ, which is important for mitochondrial replication. Depleted levels of mtDNA can lead to defects in electron transport chain function and oxidative phosphorylation causing a reduction in ATP generation and impaired energy production that may result in reduced resorptive capacity for ions and other molecules (e.g. phosphate and glucose), especially in the highly metabolically demanding and relatively hypoxic environment of the proximal tubule [34].

**Figure 1:**
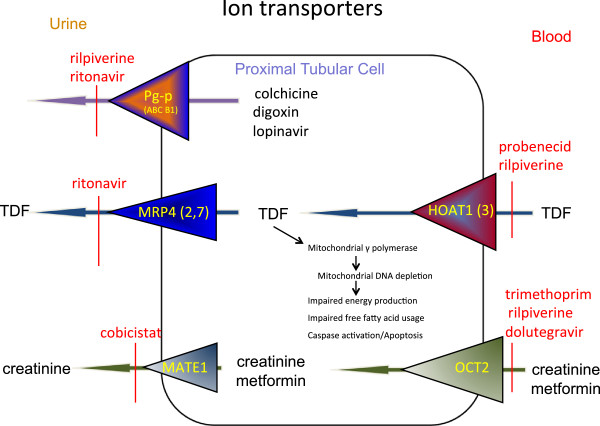
**Ion transporters involved in proximal tubular cell handling of creatinine and antiviral drugs.**

Although TFV is only a weak inhibitor of mtDNA γ-polymerase [35] the presence of a number of factors may combine to increase the risk of TFV tubulopathy in some HIV-infected individuals. Of note however, is the fact that the HIV virus itself causes mtDNA depletion, and this could be relevant when considering hypophosphataemia, including patients not on ART. TFV enters proximal tubular cells across the basolateral membrane via organic anion transporters (OAT1 and OAT3), and exits the tubule across the apical membrane via multidrug resistance protein transporters (MRP4 and possibly MRP2) coded for by the ABC cassette genes [36] (Figure 1). Co-administration of antiretroviral drugs that are also processed by these transporters (potentially affecting efflux) including ritonavir (MRP2) and didanosine (OAT1), have been implicated in many of the reports of TDF-related Fanconi syndrome. In a review of 164 cases received by the FDA Adverse Reporting System, 74% of patients were also taking a ritonavir-boosted protease inhibitor and 43% were taking didanosine (now rarely used) [37].

Declining glomerular filtration due to age or co-existing CKD can result in a shift towards increased tubular secretion of TFV and other factors such as low body weight, may increase plasma TFV levels, have also be shown to be risk factors for TDF-related tubulopathy. Finally polymorphisms in genes associated with tubular transporter proteins may affect the flux of TFV into and out of proximal tubular and may help to explain why tubulopathy occurs in only some individuals, but this data is some way from being clinically useful [38–41].

### Cobicistat

The inclusion of cobicistat in the recently introduced co-formulated tablet Stribild® (cobicistat + elvitegravir + emtricitabine + TDF), adds an extra dimension to the renal monitoring in patients receiving this TDF-based single tablet regimen. Cobicistat, which has no intrinsic anti-HIV activity, is a potent cytochrome P450 3A (CYP3A) inhibitor and is used as a pharmacoenhancer to boost and maintain the therapeutic plasma concentration of the HIV integrase inhibitor elvitegravir over the 24-hour dosing interval. Cobicistat also reduces proximal tubular secretion of creatinine by inhibiting cation transporters (particularly MATE 1) in a manner similar to cimetidine or trimethoprim. This may have implications on other drug metabolism. It does however have the potential to interact with other drugs that are metabolised by CYP3A (e.g. azol anti-fungals, warfarin, salmeterol and some statins) or those excreted by MATE1 (metformin in particular) [42]. Cobicistat leads to a modest, rapid (days) and reversible, increase in SCr, and therefore an apparent reduction in eGFR, but not GFR measured by iohexol clearance [43]. In the phase III registration studies (GS102/103) these studies involved >1400 treatment naïve patients followed to 144 weeks, the increase in SCr concentration (mean ~12.4 ± 11.5 μmol/L) stabilised after 2–4 weeks^76^. This baseline change needs to be considered when monitoring renal function in patients receiving cobicistat in combination therapy. We suggest that an immediate increase of SCr from baseline <35 μmol/L (which is taken from the mean +2 SD of the Stribild trials) be considered a range that would capture ~98% of the expected increases in patients with normal renal function and does not require additional investigation providing it is stable at this level.

### Rilpivirine

Rilpivirine is a non-nucleoside reverse transcription inhibitor, which is minimally excreted by the kidney. A small increase in serum creatinine (~8.8 μmol/L =0.1 mg/dL in the TDF backbone regimen) has been noted in studies [44, 45] and it too has been shown to inhibit a number of transporters including the organic cation transporter protein 2 (OCT2) and p-glycoprotein (pg-p) [46].

The former inhibits tubular secretion of creatinine, as cystatin C measurements of renal function are unaffected. However cystatin C based equations appear to be less accurate in patients with HIV taking ART [47, 48].

### Dolutegravir

Dolutegravir is an HIV integrase inhibitor, again largely not excreted by the kidney and again inhibiting OCT2 [49] resulting in a small rise in creatinine (by ~7.6 μmol/L in normal individuals [50]) without affecting GFR by iohexol clearance [51].

### Tenofovir in HBV

Compared to the situation in HIV, the incidence of tubulopathy with TDF when used in chronic hepatitis B (HBV) appears to be lower, although two cases of reversible Fanconi Syndrome have recently been reported [52]. This may simply reflect ascertainment or reporting bias as experience of TDF use in HBV therapy is relatively small compared to use in HIV. However there may be key differences in the HBV-infected compared to the HIV-infected population, for example HIV-infected patients are more likely to be taking concomitant nephrotoxic drugs and, unlike HIV, the HBV does not appear itself to affect mitochondrial function [53]. In addition HBV is known to be associated with glomerulonephritis with [54], or without [55] evidence of viral replication which often responds to viral suppression and that may in fact improve renal function.

Reporting of renal function (eGFR, SCr and serum phosphate, but not proteinuria) in both the long-term follow-up of the TDF HBV clinical trials in abstract form (7 years to date) and at least five prospectively-followed cohorts of patients with chronic hepatitis B (both treatment-naïve and -experienced) have revealed generally fewer changes in creatinine despite the latter cohort having a number of risk factors for renal disease.

The European Association for the Study of the Liver (EASL) 2012 management guidelines recommend only that “Renal function should be monitored during antiviral treatment” [56]. Similarly the Asian Pacific consensus recommends “close observation on proximal tubular injury and bone toxicity must be maintained” [57]. In contrast the Gastroenterology Society Australia [58] and American Association for the Study of the Liver (AASLD) [59] suggests checking the serum creatinine every 12 weeks for patients on adefovir or tenofovir. Whilst awaiting further work in this area and since viral parameters are usually being measured 3–6 monthly, we encourage the addition of a serum creatinine estimation at these time points. However we feel it would be reasonable to check the same parameters (eGFR, plasma phosphate, uPCR and for glycosuria) at least yearly in patients on TDF.

### Renal monitoring

In 2005, the Infectious Diseases Society of America recommended that all patients recently diagnosed with HIV infection should be screened for kidney abnormalities including SCr for calculation of eGFR and urinalysis dipstick for proteinuria, with annual screening for at-risk patients [60]. The more recent US DHHS suggest basic chemistry 3–6 monthly (Serum Na, K, HCO_3_, Cl, BUN, creatinine) and glucose (preferably fasting), with a suggestion that phosphorus levels should be taken in patients on TDF [18]. The more recent European AIDS Clinical Society guidelines recommend 3–12 monthly monitoring of eGFR and annual dipstick analysis for proteinuria with more frequent monitoring in high-risk groups for CKD. The Australasian Society for HIV Medicine commentary on the US DHHS guidelines recommends urinalysis (6 monthly) and electrolyte monitoring (including serum phosphate) in patients prescribed TDF in addition to routine renal monitoring of HIV-infected patients; again with more frequent monitoring in those with an increased risk of renal insufficiency.

### TDF HIV renal management algorithm

**Figure 2:**
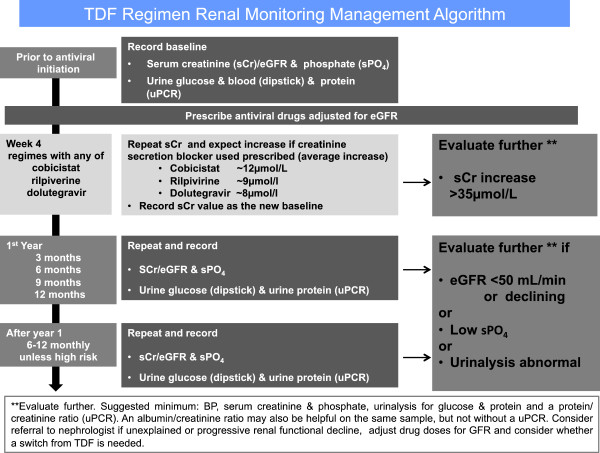
**Schematic of suggested renal monitoring when starting drugs which are potentially nephrotoxic.**

Given the central role of TDF-based therapy and the potential for tubulopathy associated with TDF, we suggest active monitoring to maximise the drug’s continued utility. These suggestions are outlined in the algorithm in Figure 2. It is important to note that this algorithm complements the *Kidney Health Check*^7^ (SCr (and thus also eGFR), albumin/creatinine ratio (uACR) and blood pressure) recommended every 1–2 years for patients with diabetes, hypertension, obesity (BMI ≥ 30), established CVD, smokers, those with a family history of CKD, and those of Aboriginal or Torres Strait Islander origin. This is in order to detect subclinical renal disease and to identify those patients with a higher cardiovascular risk or the small cohort who may go on to develop declining renal function. We suggest such monitoring is also appropriate for those who are on a non –TDF regimen. The key differences for patient on TDF based regimens is that urine total protein-to-creatinine ratio (uPCR) should be used, rather than uACR, and the additional measurement of a serum phosphate level. The measurement of blood pressure is important for detecting cardiovascular and renal risk generally. Patients who may be at high risk of developing renal dysfunction include those;

- With co-infection with viral hepatitis
- Concomitant nephrotoxic medication (especially non-steroidal anti-inflammatory drugs (NSAID))
- With diabetes
- Who are Aboriginals, Torres Straight islanders or black Africans
- Who have a family history of renal disease
- With hypertension
- With pre-existing cardiovascular or renal disease
- With uncontrolled or untreated HIV
- With a low BMI (<18.5)

### GFR estimates

In Australia, the Chronic Kidney Disease Epidemiology Collaboration (CKD-EPI) formula has largely replaced the Modification of Diet in Renal Disease (MDRD) equation for calculating eGFR [61]; CKD-EPI helps prevent underestimates of GFR particularly at the higher ranges of GFR [62]. Both equations have shown a reasonable degree of agreement in stratifying baseline eGFR in HIV positive population, although a recent publication suggests that CKD-EPI best approximates measured GFR in this group [63]. The differences between CKD-EPI eGFR and Cockcroft-Gault (CG) formula estimations of creatinine clearance are very modest (3.2 (IQR −0.6 to 7.4 mL/Min/1.73 m^2^)) and typically have equal precision. Although recommendations for dose adjustment are typically quoted as creatinine clearance (CrCl), we suggest using the CKD-EPI formula and in this context, and this can be used interchangeably with CrCl for TDF dose modification [64]. Cystatin C based equations cannot be recommended for renal monitoring in HIV positive individuals because its generation is affected by HIV itself and a number of antivirals [47, 48, 65].

### Abnormal baseline tests

If patients have abnormal test results at baseline, treatable reasons for kidney disease should be excluded and the patient’s risk of CVD assessed and managed with lifestyle modification and medication, as appropriate, to control hypertension (ACE inhibitor or angiotensin II receptor blockers as first line therapy), dyslipidaemia and hyperglycaemia. Nephrotoxic medications should be avoided and medication doses adjusted for renal function. The prescriber should consider carefully the appropriateness of commencing TDF, which may require a dosing interval adjustment (as detailed in the product information)^75^ in all patients with CrCl/eGFR < 50 (see Table 1). Stribild should not be initiated in patients with eGFR/CrCl < 70 mL/min/1.73 m^2^ and subsequently if the CrCl falls to < 50, it should be discontinued because the required dose interval adjustments are not possible using this fixed dose combination tablet.

**Table 1:** **TDF dose reduction strategies**

Creatinine clearance	Dosing interval for TDF 300 mg
(or eGFR – CKD-EPI)	
>50	Every 24 hrs
30-49	Every 48 hrs*
10-29	Every 72–96 hrs*
Haemodialysis	After dialysis every 7 days or after ~12 hrs of dialysis*

*Consider whether TDF is the appropriate antiviral.

### Proteinuria

A reduction in tubular ATP production may also allow enhanced urinary loss of low molecular weight proteins (e.g. ß2-microglobulin and retinol binding protein) which are usually freely filtered but reabsorbed by active processes involving megalin and cubilin. In contrast to glomerular disease, where large proteins such as albumin, are lost in the urine, the glomerular filtration barrier in TDF-associated renal injury is often relatively intact. This means that checking the urine for albumin alone (as recommended in the Kidney Health Australia (KHA) guidelines for detection of renal disease) may miss proteinuria due to tubular dysfunction. We recommend the use of uPCR since this will detect all urinary protein, not simply albumin. Thus glomerular disease (which may cause predominantly albuminuria) and/or tubular proteinuria (which may cause low molecular weight proteinuria) will both be detected if present [66]. A ratio of uACR to uPCR <0.4 has been suggested as a useful tool to distinguish between the two in HIV positive cohorts [67] and has been found to be helpful in determining the aetiology of proteinuria [68]. Dipstick urinalysis for protein is mainly sensitive to albumin, and especially in dilute urine may miss other (tubular) proteinuria [69].

### Glycosuria

Any method for detecting urinary glucose is valid and a dipstick is a good test. If the patient is diabetic then the test should be performed with a blood glucose estimation to ensure normoglycaemia at the time of the test. Glycosuria is not normal under any circumstance (apart from pregnancy and the rare glucose transporter defects, or with patients on the new glut 2 inhibitors) [70]. Dipstick urinalysis is sensitive to glycosuria but can be negative in the face of high vitamin C intake [69]. New glycosuria with normal serum glucose should warrant urgent repeat and if confirmed discontinuation of TDF.

### Serum phosphate

Serum phosphate levels below the reference range can occur in diverse range of conditions including untreated HIV infection. Thus, the presence of hypophosphataemia should be confirmed on a fasting specimen and other potential causes investigated.

Moderate hypophosphataemia (0.65–0.8 mmol/L) is often transient, benign and of unknown significance. Follow up isolated hypophosphataemia would be onerous, expensive, and largely unhelpful, and simply warrants monitoring at the next routine visit with a morning fasting sample. However, if the serum phosphate falls below 0.65 mmol/L, further investigation [71] may be appropriate particularly looking for features of Fanconi syndrome triad [72] (hypophosphataemia, glycosuria with normal serum glucose, mild proteinuria). Other biochemical features may include hypokalaemia, hypouricaemia, low serum bicarbonate, low serum urate. Clinical features may be polyuria and polydipsia, lassitude and bone pains.

Conditions associated with hypophosphataemia (adapted from [68]) include;

- alcohol excess
- concomitant drugs (e.g. antacids and phosphate binders, diuretics, bisphosphonates, corticosteroids)
- the Fanconi syndrome either inherited or acquired (e.g. cisplatinum, ifosphamide, antivirals)
- rare syndromes associated with FGF23 excess inherited (e.g. X-linked hypophosphataemia) or acquired (e.g. tumour induced osteomalacia), iron infusion
- a glucose or insulin load
- those with hepatic impairment
- primary hyperparathyroidism
- malabsorption syndromes
- a respiratory alkalosis (hyperventilation)
- untreated HIV

### Reasons for nephrology referral

If the Fanconi syndrome is confirmed then TDF should be stopped and the patient changed to a non-TDF containing ARV regimen, and renal function and proteinuria followed carefully with monthly testing until these features have resolved. If no improvements in these abnormalities are apparent within a month then a nephrological review is strongly recommended. Other situations when referral to a nephrologist with an interest in HIV or specialist HIV physician include:

- eGFR < 30 mL/min
- Ongoing significant proteinuria (uPCR >50 mg/mmol)
- A consistent decline in eGFR from baseline < 60 mL/min (i.e. decline > 5 mL/min over a 6-month period, confirmed ≥ 3 separate readings)
- Glomerular haematuria with albuminuria
- CKD and difficult to control hypertension despite ≥ 3 antihypertensive agents
- Microscopic or macroscopic haematuria alone should also be referred for urine cytology, imaging and possibly cystoscopy.

If renal dysfunction is not related to a full Fanconi syndrome, but thought to be related to TDF, then a prompt switch away from TDF-based antivirals is almost always the best choice. However, it is our observation is that this switch is sometimes made where the evidence implicating TDF for the renal decline is scarce or non-existent, and often without considering alternative diagnoses. The implications are that TDF is then unavailable for future use in that individual, which significantly narrows the antiviral regimen choice. Thus the decision to stop a regimen, which is usually successfully supressing virus, is not a trivial one and should be made by weighing the evidence implicating a particular drug in the renal decline.

### Frequency of follow up

Most international guidelines suggest at least yearly follow up of renal function, which we consider the minimum frequency for good care of patients with HIV-infection. Some authors advocate testing 3 monthly in the first year and yearly thereafter [73], as those who are well with completely normal tests in the first 12 months appear to be at lower risk of nephrotoxicity. The data for TDF suggest that the risk of nephrotoxicity may increase over time in at-risk patients [14]. We therefore suggest more frequent (3–6 monthly) review of patients with identified renal disease (e.g. CKD, mild proteinuria or hypophosphatemia but without glycosuria) particularly when a dose adjustment may be needed (Figure 2). Any patient with the Fanconi syndrome should be re-tested monthly until there are improvements in biochemistry and then every 3 months until back to baseline function and referred for specialist review by an HIV physician or a nephrologist with an interest in HIV. We feel it would be sensible for high risk-patients on TDF for HBV mono-infection infection to have similar renal investigations at least yearly. Patients who are not on nephrotoxic antiviral agents may still be at higher risk of developing renal dysfunction associated with non drug related issues and ageing. Thus a yearly Kidney Health Check [7] would appear be appropriate in these patients.
